# The Strength of T Cell Receptor Signal Controls the Polarization of Cytotoxic Machinery to the Immunological Synapse

**DOI:** 10.1016/j.immuni.2009.08.024

**Published:** 2009-10-16

**Authors:** Misty R. Jenkins, Andy Tsun, Jane C. Stinchcombe, Gillian M. Griffiths

**Affiliations:** 1 Cambridge Institute for Medical Research, PO Box 139, Addenbrooke's Hospital, Hills Road, Cambridge CB2 0XY, UK

**Keywords:** MOLIMMUNO, CELLIMMUNO

## Abstract

Killing by cytotoxic T lymphocytes (CTLs) is mediated by the secretion of lytic granules. The centrosome plays a key role in granule delivery, polarizing to the central supramolecular activation complex (cSMAC) within the immunological synapse upon T cell receptor (TCR) activation. Although stronger TCR signals lead to increased target cell death than do weaker signals, it is not known how the strength of TCR signal controls polarization of the centrosome and lytic granules. By using TCR transgenic OT-I CTLs, we showed that both high- and low-avidity interactions led to centrosome polarization to the cSMAC. However, only high-avidity interactions, which induced a higher threshold of intracellular signaling, gave rise to granule recruitment to the polarized centrosome at the synapse. By controlling centrosome and granule polarization independently, the centrosome is able to respond rapidly to weak signals so that CTLs are poised and ready for the trigger for granule delivery.

## Introduction

Cytotoxic T lymphocytes (CTLs) destroy virally infected and tumorigenic targets with remarkable specificity. CTL killing is provided by the delivery of perforin and granzymes from specialized secretory granules, termed lytic granules. Upon recognition of a target cell via the T cell receptor (TCR), the centrosome (the microtubule-organizing center [MTOC] in T cells) polarizes toward the site of signaling and contacts the plasma membrane at the central supramolecular activation complex (cSMAC) of the immunological synapse (Monks et al., 1998; Stinchcombe et al., 2006). Lytic granules move along microtubules in a minus-end direction, toward the centrosome, and are delivered with great accuracy to the secretory domain, which lies adjacent to the cSMAC and within the peripheral (p)SMAC and the outer, distal (d)SMAC (Grakoui et al., 1999). Lytic proteins are released into a small secretory cleft that forms between CTL and target (Stinchcombe et al., 2001). In this way, CTLs kill targets effectively with fast and focused delivery of lytic proteins, with the centrosome playing an essential role in directing lytic granules to the immunological synapse.

The triggering of cytotoxicity is known to be sensitive and rapid. The avidity of the CTL binding to its target is influenced not only by the affinity of the TCR and peptide-MHC (pMHC), but also by the number of receptors engaged and the stability of the interaction (Konig, 2002). TCR transgenic mice with clonal T cell receptors have provided a great deal of information about the signals that control killing. Although studies have demonstrated that low numbers of engaged ligands are sufficient to elicit cytotoxicity (Purbhoo et al., 2004), it is well established that the avidity of interaction between CTLs and targets controls subsequent effector functions (Alam et al., 1996, 1999; Rosette et al., 2001; Yachi et al., 2006) and higher-avidity interactions increase levels of target cell death (Jameson et al., 1993; Koniaras et al., 1999). Precisely how TCR avidity controls the polarization and release of cytotoxic granules is not known.

OT-I mice are transgenic for a TCR that recognizes the ovalbumin peptide, SIINFEKL (OVA_257-264_), presented by H-2K^b^ (Hogquist et al., 1994). CTL responses from OT-I mice are particularly well characterized with respect to a range of altered peptide ligands with varying avidities for the transgenic TCR (Alam et al., 1999; Jameson et al., 1993; Koniaras et al., 1999; Rosette et al., 2001; Yachi et al., 2006). CTL-mediated killing decreases as TCR avidity is reduced either by decreasing concentrations of peptide or by substituting OVA_257-264_ with the altered peptide ligand G4. Position 4 is a critical residue for TCR interaction (Jameson and Bevan, 1992) and the replacement of asparagine with glycine produces a peptide, G4, that binds to the MHCI H-2k^b^ with the same affinity as OVA_257-264_, but binds the OT-I TCR with a lower affinity than OVA_257-264_. Surface plasmon resonance (SPR) and tetramer dissociation showed that G4 has a faster off-rate than does OVA_257-264_ for the OT-I TCR (Rosette et al., 2001). G4 is unable to induce sustained early TCR signaling events (Rosette et al., 2001; Yachi et al., 2006) and stimulates very low levels of target cell death compared to OVA_257-264_ (Jameson et al., 1993; Koniaras et al., 1999).

We decided to take advantage of the OT-I system to ask how different avidity interactions control the polarization of the centrosome and lytic granules to the immunological synapse by using different concentrations of OVA_257-264_ or G4 to provide interactions with different avidities. We monitored the formation of the immunological synapse and polarization of the centrosome and lytic granules. We found that OVA_257-264_ induced activation at the cSMAC, which triggered both centrosome and granule polarization. However, G4 induced only transient activation at the cSMAC, which, although sufficient to trigger centrosome polarization, was insufficient to trigger recruitment and delivery of granules at the synapse. Our results reveal that TCR avidity controls the different steps required to deliver cytotoxicity, independently, providing very fine control of CTL-mediated killing.

## Results

### Low-Avidity Interactions Are Sufficient for Conjugate and Synapse Formation Despite Poor Effector Function

**Figure 1:**
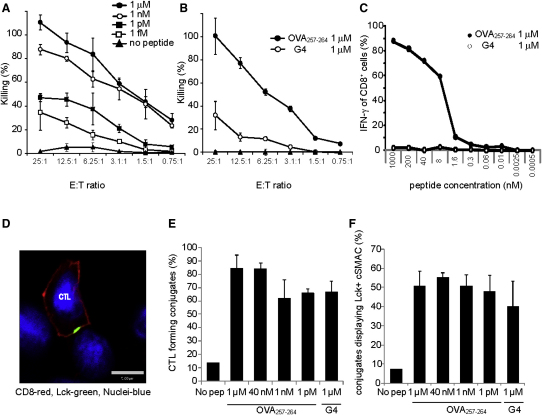
OT-I Conjugate Formation and Effector Responses to OVA_257-264_ and G4 (A and B) OT-I CTL incubated with EL4 targets, peptide pulsed with (A) differing concentrations of OVA_257-264_ peptide or with (B) 1 μM OVA_257-264_ or G4. Graphs show the mean percent cytotoxicity of triplicates ± SD for varying effector to target (E:T) ratios and are representative of four independent experiments. (C) Graph of the mean percent CTLs stained intracellularly for IFN-γ and analyzed by flow cytometry at peptide concentrations shown for G4 (open circles) and OVA_257-264_ (closed circles). (D–F) OT-I conjugated to OVA_257-264_ and G4 peptide-pulsed EL4 target cells at 37°C for 20 min, stained with anti-Lck (AlexaFluor 488; green) and anti-CD8 (AlexaFluor 546; red). An example of Lck clustering at the cSMAC (single-plane confocal image) (D); the mean percent of CTLs in conjugates (E); and the mean percent of conjugates with Lck accumulation at the immunological synapse (F) is shown (±SD, average of four experiments). Four separate experiments were carried out for each set of conditions. At least 300 conjugates were counted for each experiment, and graphs show the mean of four independent experiments. Differences were not statistically significant according to a Student's t test (p > 0.06). Scale bar represents 5 μM.

We examined the ability of OT-I CTL to induce target cell death, produce cytokine, form conjugates, and form immunological synapses in response to TCR signals of varying avidity. OT-I splenocytes were stimulated in vitro with 10 nM OVA_257-264_ for 5 days to produce activated CTLs that were used for all subsequent experiments. Cytotoxicity was determined by lactate dehydrogenase (LDH) release from targets that had been peptide pulsed with either OVA_257-264_ or G4 peptide at the concentrations shown. Decreasing concentrations of OVA_257-264_ resulted in decreasing amounts of target cell lysis, whereas unpulsed targets were not killed (Figure 1A). Although EL4 targets pulsed with OVA_257-264_ were killed very efficiently, G4-loaded targets were killed poorly (Figure 1B). Cytokine production is strongly affected by the strength of TCR signal and requires long, stable CTL-target interactions and synapse formation (Valitutti et al., 1996). No IFN-γ was detected after stimulating OT-I in the presence of 1 μM G4 peptide for 4 hr (Figure 1C), consistent with previous findings (Jameson et al., 1993).

We chose to study conjugates formed at 20 min throughout the study, in order to keep a standard set of conditions for comparing the avidity. Fixed conjugates were examined by confocal microscopy after labeling with antibodies against CD8 and lymphocyte protein tyrosine kinase, Lck, to mark the cSMAC (Figure 1D). Few conjugates formed in the absence of peptide (Figure 1E). Although there were fewer conjugates with a decrease in avidity between 1 μM OVA_257-264_ and 1 μM G4, this difference was not statistically significant when compared via analysis of variance (p = 0.14). Our data indicate that the number of conjugates formed at 20 min was unaffected at lower-avidity interactions, consistent with previous studies that show a correlation between avidity and cytotoxicity (al-Ramadi et al., 1995; Rosette et al., 2001; Yachi et al., 2006).

Next we examined cSMAC formation by investigating the clustering of Lck at the immunological synapse. Lck is a tyrosine kinase, which associates with CD8 and clusters at the cSMAC upon TCR activation (Monks et al., 1998). It provides a marker of TCR signaling and early synapse formation. In the absence of any peptide, clustering of Lck occurred in only 7.4% of conjugates, which formed very rarely (Figures 1E and 1F). However, under all conditions in which OT-I cells were stimulated with either G4 or OVA_257-264_ pulsed targets, >40% of conjugates displayed clustering of Lck and formation of a cSMAC (Figure 1F).

These results reveal that although the killing and cytokine production varied dramatically with avidity, conjugate formation and cSMAC formation occurred equally well over the range of avidities used in these experiments. This provided a system to examine the affect of avidity on downstream cytotoxic effector mechanisms.

### Src Family Kinases Are Rapidly Dephosphorylated in Low-Avidity Synapses

**Figure 2:**
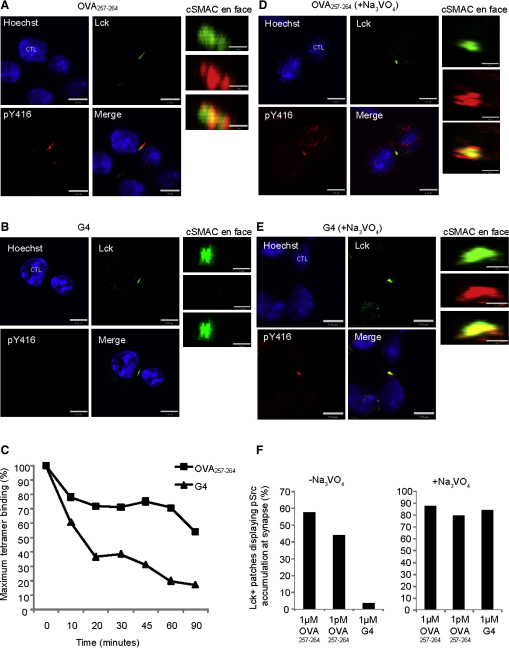
cSMAC Activation Correlates with Avidity (A and B) Target EL4 cells pulsed with (A) 1 μM OVA_257-264_ or (B) G4 peptides, conjugated to in vitro activated OT-I CTLs, stained with anti-Lck (488, green) and pY416 (546, red). Images are shown as projections through the xy plane, or xz plane reconstructed from a 1 μM section across the synapse (en face). Scale bars represent 1.5 μM. (C) Graph showing the percent maximum tetramer stained OT-I relative to time zero for OVA_257-264_-K^b^-PE, and G4-K^b^-PE tetramers over 1 hr at 37°C, detected by flow cytometry. (D and E) Conjugates prepared as in (A) and (B), but preincubated with orthovanadate. (F) The mean percent (from two experiments) of Lck^+^ staining synapses with distinct pY416 accumulation in the presence or absence of sodium orthovanadate.

Because OVA_257-264_ and G4 provide different avidity interactions, we examined T cell signaling at the cSMAC for each. CTLs were conjugated to OVA_257-264_ or G4-pulsed EL4 targets before labeling with antibodies against CD8, Lck, and phospho-Src-family kinases. The pSrc-family antibody (pY416) detects phosphorylated tyrosine 416 in Src family kinases, including the activated form of Lck (Ilani et al., 2009). Of the Src-family protein kinases, only Lck, Fyn, and Yes are expressed in T cells (http://symatlas.gnf.org; Samelson et al., 1990) and only Lck and Fyn have been shown to be able to cluster at the cSMAC (Monks et al., 1998). Conjugates were examined by confocal microscopy in the xy plane and xz plane to view the conjugates from the side and en face, respectively (Figures 2A and 2B). OVA_257-264_-pulsed conjugates showed colocalization of Lck and pY416 staining, whereas no pY416 staining was detected in G4 conjugates. A projection through the z plane of the synapse of OVA_257-264_ conjugates revealed pY416 staining at the cSMAC, colocalizing with Lck staining (Figure 2A). In contrast, no pY416 staining was detected in the majority of G4 conjugates (Figure 2F). The diameter of the cSMAC varied between 1.5 and 3 μM at the widest point in conjugates formed under any of the conditions tested.

G4 rapidly dissociates from the OT-I TCR, compared to OVA_257-264_ (Rosette et al., 2001), consistent with the idea that it occupies the TCR more transiently. To establish how great this difference in the off-rate is in conjugates formed after 20 min, we examined the dissociation of the K^b^G4-PE and K^b^ OVA_257-264_-PE tetramers from OT-I, in the presence of an antibody to H-2D^b^ and H-2K^b^ that prevents rebinding of dissociated tetramers. CTLs were detected by flow cytometry and the percentage of tetramer-bound CD8^+^ cells were plotted over time to show the rate of dissociation (Figure 2C). OT-I CTL rapidly lost G4-PE tetramer compared to OVA_257-264_-PE tetramer. At 20 min, only half the number of OT-I bound G4 tetramer compared to OVA_257-264_. This suggested that any signaling in response to G4 might be inhibited by phosphatases, such as SHP-1 (Stefanova et al., 2003) in G4 conjugates.

To investigate this possibility, we generated OT-I conjugates in the presence of sodium orthovanadate (Na_3_VO_4_), which prevents dephosphorylation of tyrosine kinases, by inhibiting phosphatases (Figures 2D and 2E). Under these conditions, pY416 staining was seen in the cSMAC in more than 75% of OVA_257-264_ and G4 conjugates (Figure 2F), colocalizing with Lck in both cases. Orthovanadate treatment did not affect amounts of pY416 staining in OT-I cells in the absence of peptide (Figure S1 available online). These results reveal that activation occurs at the cSMAC in response to both OVA_257-264_ and G4; however, activation in response to G4 is inhibited by phosphatase activity.

### pERK Activation Occurs in Response to High- and Low-Avidity Interactions

**Figure 3:**
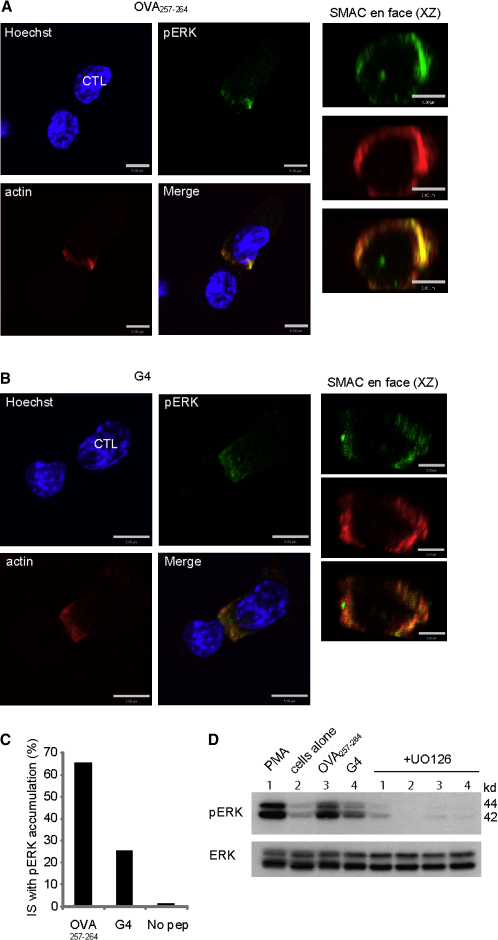
Phospho-ERK Accumulates in the dSMAC, with Actin (A and B) Immunofluorescent images of in vitro activated OT-I cells conjugated to target EL4 cells, prepulsed with either (A) 1 μM OVA_257-264_ or (B) 1 μM G4 peptides. Cells are stained with Hoechst (blue) and antibodies against pERK (AlexaFluor 488; green) and actin (AlexaFluor 546; red). Confocal projections of conjugates are shown in the xy plane (scale bars represent 5 μM) or as reconstructions across 1 μM of synapse (en face) (scale bars represent 3 μM). (C) Quantitation of percent conjugates with the centrosome polarized to the synapse, displaying pERK accumulation at the dSMAC (n = 100) from three separate experiments. (D) Immunoblot of cell lysates from OT-I CTLs stimulated with 50 nM PMA (1), untreated (2), or stimulated with 1 μM OVA_257-264_ (3) or G4 peptide (4), in the presence or absence of UO126 MEK inhibitor, probed with antibodies against pERK or total ERK. Molecular weights are shown. Representative of two separate experiments.

Previous studies have shown that Lck activation leads to recruitment of the phosphatase, SHP-1, which downregulates Lck activity. This negative-feedback loop is balanced by a positive-feedback loop controlled by phospho-ERK (pERK), which prevents SHP-1 recruitment to Lck (Stefanova et al., 2003). Furthermore, pERK has been shown to accumulate at the synapse between CTL and the antigen-presenting cell more strongly in response to OVA_257-264_ than G4 via RMAS antigen-presenting cells (APCs) (Yachi et al., 2006). In addition, pERK signaling has been implicated in granule polarization and actin remodeling in both T cells and NK cells (Chen et al., 2006; Robertson et al., 2005). For these reasons, we investigated whether pERK accumulated at the immunological synapses of both OVA_257-264_ and G4 conjugates with EL4 APCs and pERK activation with these stimuli. Confocal microscopy of conjugates revealed that pERK accumulated at the synapse of both OVA_257-264_ and G4 conjugates (Figures 3A and 3B), and the pattern of staining was distinctive when viewed in the xz plane (Figures 3A and 3B). pERK staining formed a ring, which overlapped with actin, in the dSMAC. Occasionally, a small patch of pERK was observed within this ring. The specificity of antibody staining was confirmed by preincubation with the mitogen-activated protein kinase kinase (MEK) inhibitor, U0126, which abolished staining (Figure S2). Although pERK staining accumulated in the dSMAC in both OVA_257-264_ and G4 conjugates, the intensity of staining appeared weaker in G4 compared to OVA_257-264_ conjugates (Figures 3A and 3B). pERK activation was assessed both by quantitation of conjugates and by immunoblotting of cell lysates. Only 25% of conjugates showed pERK accumulation at the synapse after G4 stimulation compared to 65% with OVA_257-264_ (Figure 3C). Furthermore, immunoblotting on cell lysates stimulated with OVA_257-264_ showed strong ERK activation, almost comparable to stimulation with the phorbol ester, phorbol 12-myristate 13-acetate (PMA), and well above the basal amount observed in OT-I in the absence of peptide (Figure 3D). Stimulation with G4 showed weak ERK activation, which was nevertheless above the basal level observed in control cells in the absence of peptide. Addition of the MEK inhibitor U0126 largely ablated the pERK signals, although the total amounts of ERK in the samples were comparable. These results implied a close association between actin and pERK and demonstrated that even the low-avidity peptide, G4, gave rise to low ERK activation that accumulated in the dSMAC of the synapse. Moreover, these results showed that actin clearance from its initial accumulation across the synapse to form the ring of the dSMAC occurred in both OVA_257-264_- and G4-stimulated synapses.

**Figure 4:**
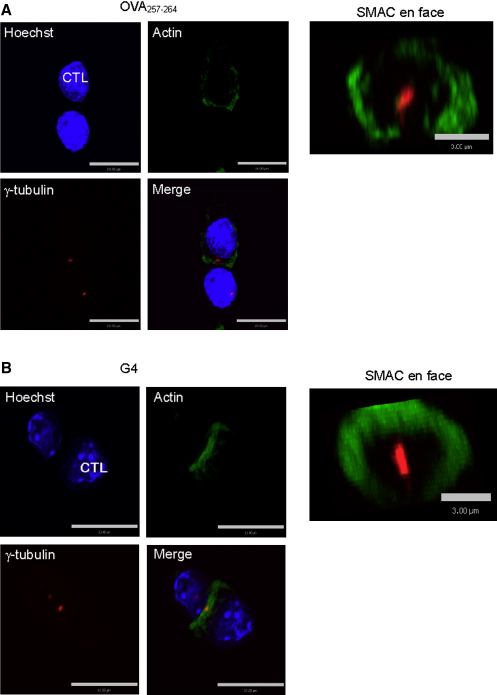
Actin Clearance and Centrosome Polarization at the Immunological Synapse of OVA_257-264_ and G4 Conjugates OT-I conjugates with EL4 pulsed with (A) 1 μM OVA_257-264_ or (B) G4. Conjugates are stained with Hoechst (blue), actin (AlexaFluor 488; green), and γ-tubulin (AlexaFluor 546; red). Images show a confocal projection through the xy axis of conjugates (scale bars represent 10 μM), and a 1 μM projection through the z axis of the SMAC (en face) (scale bars represent 3 μM).

Our previous studies have shown that, upon TCR activation, actin clears to form the dSMAC as the centrosome moves forward to dock at the plasma membrane, suggesting that the two events may be linked (Stinchcombe et al., 2006). Our findings that actin clearance could occur in both OVA_257-264_ and G4 conjugates suggested that centrosome polarization might also be occurring in both sets of conjugates. Antibody staining for both actin and γ-tubulin revealed that actin cleared into a characteristic ring with the centrosome (labeled with γ-tubulin) contacting the plasma membrane in the center of this ring in both OVA_257-264_ and G4 conjugates (Figure 4). This is particularly clear in xz reconstruction over 1 μM across the synapse.

### Both Strong and Weak TCR Signals Elicit Efficient Centrosome Polarization to the cSMAC

**Figure 5:**
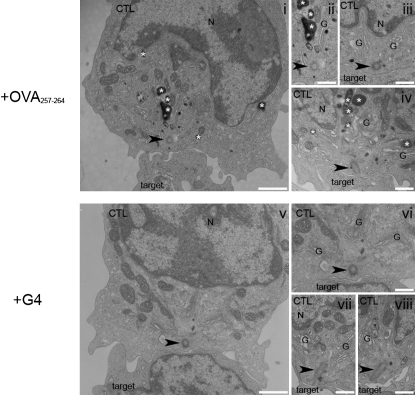
Polarization of the Centrosome in CTL-Target Cell Conjugates Low- (i, v) and high- (ii–iv, vi–viii) power electron micrographs showing thin (50–100 nm) sections of OT-1-EL4 conjugates with 1 μM OVA_257-264_ (i–iv) or G4 (v–viii). OT-I (CTL), lytic granules (white asterisks), polarized centrioles (arrowheads), Golgi complex (G), nucleus (N). Scale bars represent 1 μm (i, v) or 0.5 μm (ii–iv, vi–viii).

**Figure 6:**
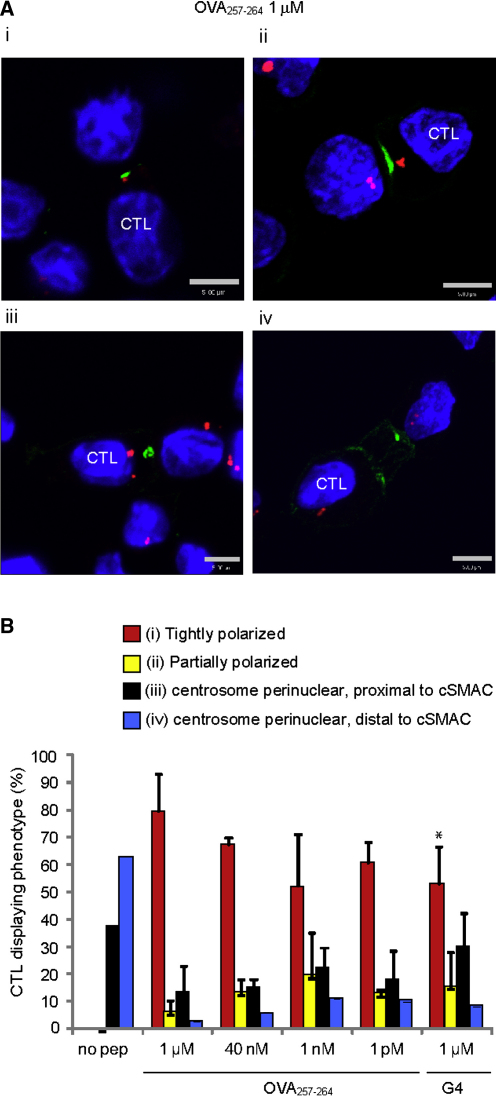
Quantitation of Centrosome Polarization (A) OT-I CTLs conjugated to target EL4 cells, pulsed with 1 μM OVA_257-264_ peptide, stained with Hoechst (blue), and labeled with antibodies against Lck (Alexa Fluor 488; green) and γ-tubulin (Alexa Fluor 546; red). Examples of centrosome localization illustrating different phenotypes are shown: (i) centrosome tightly polarized to the cSMAC, (ii) centrosome partially polarized to the cSMAC, (iii) centrosome perinuclear, proximal to the cSMAC, or (iv) centrosome perinuclear, distal from the immunological synapse (scale bars represent 5 μM). (B) Percentages of conjugates displaying each phenotype shown in (A), with varying peptide concentrations. Graph displays the mean percent OT-I with each phenotype from three or more independent experiments ± SD. Statistical significance between OVA_257-264_ 1 μM and G4 1 μM was determined by Student's t test, where p = 0.01 (asterisk). A minimum of 247 conjugates were counted for each set of conditions and the graphs show the mean scores of four independent experiments.

We have previously shown that the centrosome not only polarized to the synapse but also moved right up to the plasma membrane (Stinchcombe et al., 2006). To confirm whether or not avidity affected the tightness of polarization, we examined the synapses formed between OT-I CTLs and targets pulsed with either 1 μM OVA_257-264_ or G4 at high resolution via electron microscopy (EM) (Figure 5). Low-power images of OVA_257-264_-stimulated OT-I (Figure 5, i) showed the centrosome, seen in cross-section through one centriole (indicated by an arrowhead), polarized to the plasma membrane, distant from the nucleus. These thin sections are consistent with 3D images taken of conjugates with the nucleus stained with Hoechst (compare to Figure 6), and with previous studies in other types of CTL (Stinchcombe et al., 2006). High-power images of transverse sections through the barrel of one centriole (Figure 5, ii–iv) revealed that both the centrosome and Golgi stacks (G) were clearly visible polarizing right up to the plasma membrane. Consistent with previous studies, the tightness of polarization of both the centrosome and Golgi stacks relative to the membrane varied between conjugates, with some showing the centriole at the plasma membrane and others, showing the centriole slightly back from it (compare to Stinchcombe et al., 2006). These differences are likely to reflect images of cells captured at different stages of interaction (i.e., polarization, killing, retraction) with their targets at the time of fixation. Electron-dense lytic granules were seen in the OVA_257-264_-pulsed OT-I cell close to the centrosome (Figure 5; i, ii, and iv) as well as near the nucleus (Figure 5, i). Similar instances of tight centriole and Golgi polarization were also found in OT-I-G4 conjugates, indicating that lower avidity did not affect the tightness of either centriole or Golgi polarization (Figure 5; vi–viii). Transverse sections through the barrel of one centriole revealed how closely associated the centrosome is with the plasma membrane and suggests that it contacts the plasma membrane directly, consistent with immunofluorescent images (Figure 5; Stinchcombe et al., 2006).

In order to quantitate centrosome polarization in response to avidity, we examined the position of the centrosome (γ-tubulin labeling) relative to the cSMAC (Lck labeling) in conjugates formed with different concentrations of OVA_257-264_ or G4 (Figure 6). The centrosome is dynamic and usually associated with the nucleus (perinuclear). In migrating T cells, the centrosome is localized on the trailing edge of the nucleus (Burkhardt et al., 2008), but on synapse formation reorients toward the contact site (Kupfer et al., 1985). In a fully polarized CTL, the centrosome migrates right up to and docks at the plasma membrane next to the cSMAC (Stinchcombe et al., 2006). By using immunofluoresence microscopy, we examined OT-I CTLs conjugated to EL4 target cells loaded with either OVA_257-264_ or G4. Conjugates were classified into four groups (as illustrated in Figure 6A) based on the position of the centrosome within the cell: (i) centrosome tightly polarized at the plasma membrane, next to the cSMAC, (ii) partially polarized between the nucleus and the cSMAC, (iii) perinuclear and proximal to the cSMAC, or (iv) perinuclear and distal from the cSMAC (Figure 6A). In conjugates formed in the absence of peptide, the centrosome was always perinuclear and never reached the cSMAC (Figure 6B). However, in conjugates formed in the presence of OVA_257-264_ or G4, the centrosome was tightly polarized to the cSMAC in the majority of cases. The mean numbers varied, with 80% of OVA_257-264_ conjugates polarizing the centrosome compared with 53% for G4. A Student's t test comparing the two groups gave a value of p = 0.01, suggesting that this difference is statistically significant. This suggests that decreasing avidity may reduce the proportion of conjugates in which the centrosome polarizes and may contribute to the reduction in killing seen in response to G4. However, in spite of the fact that the centrosome polarized in the majority of G4 conjugates, there is virtually no killing of target cells observed under these conditions (Figure 1). We therefore examined granule polarization in response to G4 versus OVA_257-264_ stimulation.

### The Strength of TCR Signal Controls Lytic Granule Recruitment to the Immunological Synapse

**Figure 7:**
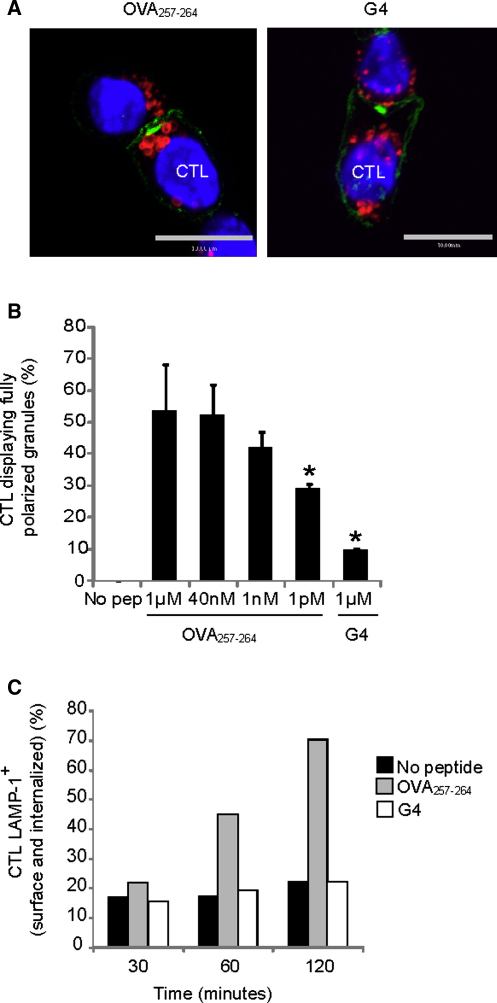
Granule Polarization and Release by OVA_257-264_ and G4 Conjugates (A and B) OT-I CTLs conjugated to target EL4 cells, pulsed with different concentrations of OVA_257-264_ or 1 μM G4 peptide, and labeled for confocal microscopy with Hoechst (blue, nuclei) and antibodies against LAMP-1, to identify granules (red), and Lck to label the cSMAC (green). Representative immunofluorescent projections of OVA_257-264_ (left) and G4 (right) conjugates are shown (A). The mean percent of Lck^+^ conjugates displaying fully polarized granules to the immunological synapse was quantitated (B) and displayed as the mean percent of more than three independent experiments ± SD (n > 163). Statistical significance, p ≤ 0.05 (asterisk) was determined with a Student's t test. Scale bars represent 10 μM. (C) LAMP-1 degranulation assay showing percent LAMP-1^+^ OT-I, after incubation with EL4 cells pulsed with no peptide (black bars), 1 μM OVA_257-264_ (gray bars), 1 μM G4 (white bars) for 30, 60, or 120 min, in the presence of LAMP-1-PE. Representative of three separate experiments.

Lytic granule polarization to the synapse was compared in conjugates formed with OVA_257-264_- and G4-pulsed targets. Immunofluorescence microscopy was used to identify lytic granule localization relative to the cSMAC by using antibodies against Lck (cSMAC) and the lysosomal membrane protein, LAMP-1 (CD107a) (Figure 7), a transmembrane protein that localizes to lytic granules (Peters et al., 1991). Because LAMP-1 staining detected lysosomes in both targets and CTLs, CD8 staining was used to identify CTLs, and in addition the lytic granules of OT-I CTLs were easily distinguished by their larger size (Figure 7A). Although the majority (54%) of OVA_257-264_ conjugates showed granules clustered tightly by the cSMAC, the majority of G4 conjugates (90%) showed the granules dispersed and distant from the cSMAC (n ≥ 1115 for each group) (Figure 7B). Decreasing concentrations of OVA_257-264_ led to a decrease in the proportion of conjugates displaying fully polarized granules, with a mean of 29% of conjugates with fully polarized granules with 1 pM OVA_257-264_ compared with 54% at 1 μM OVA_257-264_. These results suggest that the strength of the TCR-target interaction controls the efficiency of granule polarization. The granule polarization results correlated with the marked decline in cytotoxic effector function shown in Figure 1 and suggested that the loss of cytotoxicity associated with G4 interactions was likely to reflect decreased CTL degranulation. In order to address this, we measured the ability of the OT-I to release lytic granules in response to OVA_257-264_ and G4 (Figure 7C). CTLs were stimulated with either G4 or OVA_257-264_ peptide and the surface mobilization of LAMP-1 from lytic granule membranes was detected by flow cytometry. In these assays, α-LAMP1-PE antibodies were present in the media throughout the duration of the assay, and any LAMP-1 that appears on the surface becomes labeled, even when subsequently endocytosed. This provides a measure of degranulation. LAMP-1 labeling increased only in OVA_257-264_-stimulated CTLs, but not in G4-stimulated cells where LAMP-1 labeling remained at the background count of cells without peptide. Assaying LAMP-1 labeling at 30, 60, and 120 min showed an increase in the percentage of CTLs labeled with LAMP-1-PE, indicating that an increasing number of OVA_257-264_-stimulated CTLs degranulated with time. However, G4-stimulated cells showed no increase compared to unstimulated controls at any of the three time points assayed. These results show that TCR avidity affects the proportion of cells that can mobilize granules to the synapse.

## Discussion

Our previous studies have shown that the centrosome plays a key role in delivering lytic granules to the immunological synapse. The centrosome is highly dynamic, scanning the synapse (Kuhn and Poenie, 2002), before docking at the cSMAC within the synapse (Stinchcombe et al., 2006). Lytic granules, traveling along microtubules in a minus-end direction, are delivered to the synapse around the cSMAC, forming the secretory domain (Stinchcombe et al., 2001, 2006). These findings suggested that centrosome docking could be the decisive step in determining whether a CTL kills its target. Or, phrased more colloquially, that centrosome docking might be the “point of no return.”

In order to address this question, we examined the polarization of both centrosome and granules in response to different strengths of TCR signals generated by interactions with different avidities. We found that over the range of avidities used, there was little effect on conjugate or cSMAC formation. However, whereas high-avidity interactions produced strong cSMAC and pERK activation, in low-avidity interactions only weak ERK activation was detected. What was surprising was that the centrosome polarized to the cSMAC in response to both high- and low-avidity interactions even though low-avidity interactions resulted in very poor killing of targets. The difference lay in the ability of high-avidity interactions to trigger granule polarization whereas low-avidity interactions were unable to do so. These results show that centrosome and lytic granule polarization are independently regulated in response to the strength of TCR signaling and suggest that although the centrosome responds readily to TCR signals, the granules require a higher threshold of signaling in order to be recruited to the synapse.

In order to compare the effects of changes in avidity alone, we tried to keep other parameters the same. All experiments were carried out with in vitro activated OT-I, and all conjugates were prepared after 20 min. Although other studies have shown variation in the numbers of conjugates formed with OVA_257-264_ and G4 over time (Yachi et al., 2006), the numbers of conjugates formed at 20 min did not vary substantially with different concentrations of OVA_257-264_ or G4. In each of our studies, we quantitated at least 200 conjugates under each set of conditions and carried out the same experiment at least four separate times. With these experimental conditions, we were able to compare the mean values statistically and found no significant difference between the number of conjugates formed or the number of conjugates that formed cSMACs with different avidity interactions. This provided an ideal scenario for analyzing what happened to synapse activation and polarization of the cytoskeleton and secretory granules in the conjugates formed because the number or conjugates with synapses were virtually identical between different conditions.

Whether the cSMAC is the site of signaling has been controversial. Early signaling events have been shown to occur in peripheral microclusters that coalesce into the cSMAC (Varma et al., 2006) where signaling is terminated as the TCR is internalized and degraded (Alcover and Alarcon, 2000; Lee et al., 2002; Liu et al., 2000). However, more recent experiments have shown signaling in the cSMAC at later time points and lower antigen doses (Cemerski et al., 2008). Our findings reveal that the cSMAC was activated in both high- and low-avidity CD8 synapses. However, Lck signaling was apparent in G4-stimulated conjugates only after treatment with sodium orthovanadate to inhibit phosphatases. This implies that signaling occurred in G4 synapses but was rapidly turned off by phosphatases. These observations are consistent with previous findings showing that Lck-mediated signaling leads to recruitment of the phosphatase SHP-1 that downregulates Lck-mediated signaling (Stefanova et al., 2003). Strong agonists have been found to sustain signaling by activation of ERK, which prevents SHP-1 recruitment. Consistent with this idea, we found strong activation of ERK by OVA_257-264_, but only weak activation of ERK by G4, which elicits a much weaker avidity interaction (Jameson and Bevan, 1992). Importantly, we found that pERK clusters at the dSMAC of the synapse in response to both G4 and OVA_257-264_. It seems likely that the reduced activation in response to G4 might be connected with the peptide's fast off rate from the OT-I TCR (Jameson et al., 1993), which suggests that G4 binds the TCR much more transiently than does OVA_257-264_. At the time point used in this study, there is a big difference in the dissociation of G4 compared to OVA_257-264_ from the OT-I TCR. Consistent with these differences in signaling, early activation events, including CD69 upregulation and Jun activation, have been shown to be delayed in naive OT-I cells stimulated with G4 compared with OVA_257-264_ (Rosette et al., 2001), and signaling has been found to be reduced and delayed in response to G4 (Yachi et al., 2006).

Given the role of ERK activation in maintaining cSMAC signaling, it was of interest to examine this pathway in our system. Several studies have shown an accumulation of pERK at the synapse of CTLs and NK cells (Giurisato et al., 2009; Yachi et al., 2006), although its precise location within the synapse has not been characterized. Our finding that pERK colocalizes with actin is intriguing because ERK activity has been implicated not only in MTOC and granule polarization in natural killer cells and CTLs (Chen et al., 2006; Jiang et al., 2002; Li et al., 2008; Robertson et al., 2005), but also in actin remodeling via cortactin (Martinez-Quiles et al., 2004). Our previous data demonstrated that the clearance of actin from the synapse correlated with the movement of the centrosome to the plasma membrane and suggested that the centrosome is brought forward to the plasma membrane as actin clears. The adaptor protein ADAP (also known as SLAP-130 or Fyb) recruits the minus end motor dynein to this ring that can reel in the MTOC or centrosome (Combs et al., 2006; Poenie et al., 2004). Interestingly, ADAP has also been shown to control TCR-mediated ERK activation (Boerth et al., 2000). More recently, the MAP kinase scaffold protein, KSR1, which enhances ERK phosphorylation, has also been shown to be required for MTOC and granule polarization (Giurisato et al., 2009). Our finding that pERK accumulation correlates with centrosome localization at the cSMAC supports a role for ERK in centrosome polarization. All of these findings are in keeping with a model in which even transient TCR activation is sufficient to activate ERK. Activated pERK is then able to play a role in actin remodeling and may be involved in clearance of the actin ring. This, in turn, would result in polarization of the centrosome to the cSMAC. The centrosome is then poised for the granules to move. Our finding that the centrosome is able to respond to low-avidity signals explains the rapid oscillation of the MTOC observed when CTLs encounter two potential targets (Kuhn and Poenie, 2002).

Our results demonstrate that centrosome and granule polarization are separately controlled and that although the centrosome responds readily to signaling, the granules do not. Granule polarization requires sustained activation at the cSMAC. The exact nature of this difference in signal is not clear, but one interesting possibility arises from recent studies on PKCδ null mice. CTLs from these mice show decreased killing because of loss of granule polarization, although centrosome polarization was not examined (Ma et al., 2008). Other studies that have focused on the role of signaling pathways that mediate polarization of granules to the synapse have implicated both ERK and PI3K pathways (Robertson et al., 2005). Because many of the signaling pathways are interconnected, with multiple roles, and are often essential for CTL development, it is very likely that multiple signaling proteins play a role in granule polarization.

## Experimental Procedures

### Generation of Mouse CTL and Cell Culture

Female C57BL/6 (B6)-OT-I naive spleens were stimulated with 10 nM OVA_257-264_ peptide for 3 days in RPMI medium 1640 supplemented with 10% FCS, 50 μM β-mercaptoethanol, 10 U/mL human recombinant IL-2, L-glutamine, sodium pyruvate, and 50 U/mL penicillin and streptomycin (GIBCO). After 2 days, cells were washed and seeded into fresh media daily. Target H-2^b^ EL4 cells were maintained in Dulbecco's Modified Eagle's Medium (DMEM) supplemented with 10% FCS and L-glutamine.

### Antibodies and Reagents

For the immunofluorescence studies, reagents were obtained from the following: mouse anti-actin (AC-40), rabbit anti-actin, and rabbit anti-γ-tubulin (Sigma-Aldrich); mouse anti-mouse Lck (3A5) (Millipore); rat anti-mouse CD8 (YTS192) (H. Waldmann, Oxford University); rat anti-mouse CD107a (LAMP-1, 1BD4) (Developmental Studies Hybridoma Bank, IA); mouse-anti-Phospho-ERK (pT202/pY204) (BD Biosciences); rabbit polyclonal anti-mouse-phospho-Src-family (pY416) (Cell Signaling); and Hoechst 33342 (Invitrogen). All secondary Alexa Fluor antibodies (excited at 405, 488, 546, and 633 nm) were from Invitrogen. For the degranulation assays, rat anti-mouse CD107a-PE (1D4B) and rat anti-mouse CD8α-PerCP-Cy5.5 (53-6.7) were from PharMingen and BD Biosciences. For tetramer dissociation experiments: OVA_257-264_/K^b^-SA-PE (streptavidin-phycoerythrin) and G4/K^b^-SA-PE tetramers (Beckman Coulter); and mouse anti-mouse-H-2D^b^/K^b^ (28-8-6) was obtained from PharMingen, BD Biosciences. OVA_257-264_ [SIINFEKL] and G4 [SIIGFEKL] peptides were obtained from AnaSpec, Cambridge Biosciences.

### Immunofluorescence and Confocal Microscopy

EL4 cells were pulsed with various concentrations of either SIINFEKL (OVA_257-264_) or SIIGFEKL (G4) peptide at 37°C for 1 hr before washing three times in RPMI. In vitro activated OT-I CTL cells (day 6 after stimulation, unless otherwise indicated) were also washed in RPMI before CTL and target cell pellets were resuspended to a final concentration of 4 × 10^6^ cells/ml. CTLs and targets were mixed 1:1 and incubated in suspension for 5 min, before diluting to 10^6^/ml and aliquoting onto glass multiwell slides, and incubated for a further 15 min at 37°C to adhere to the glass. In some experiments (Figure 2; Figure S1), cells were conjugated in presence of 1 mM sodium orthovanadate (Na_3_VO_4_). Samples were fixed with ice-cold methanol for 5 min and washed six times in PBS before incubating for 1 hr at room temperature in blocking buffer (PBS, 1% BSA [Sigma-Aldrich]). Samples were incubated with primary antibodies in PBS, 0.2% BSA for 1 hr at room temperature, or overnight at 4°C and washed extensively in PBS, 0.2% BSA before adding secondary antibodies for 40 min at room temperature. Nuclei were stained with Hoechst (1:10,000) in PBS for 5 min before mounting with number 1.5 coverglass and Mowiol. Samples were examined with a Zeiss Confocal LSM510 microscope, with lasers exciting at 405, 488, 543, and 633 nm, with the 100× objective. Final images are displayed as projections of 3D stacks unless otherwise indicated. xz reconstructions were processed with Volocity software (Perkin-Elmer).

### Cytotoxicity and Degranulation Assays

Cytotoxicity was examined with the CytoTox 96 Non-Radioactive Cytotoxicity Assay (Promega). Target EL4 cells were pulsed with peptide at 37°C for 1 hr, washed three times, and resuspended in phenol red-free RPMI, 2% FBS (killing assay media), at 10^5^ cells/mL in a round-bottom 96-well plate. Activated CTL were added at titrated effector:target (E:T) ratio and plates were incubated at 37°C for 4 hr. The absorbance of the supernatants at 490 nm determined the release of lactate dehydrogenase (LDH) and percent cytotoxicity. For degranulation assays, effector CTL and targets were mixed at a ratio of 1:1 and incubated in 96-well plates at 37°C, in the presence of CD107a-PE (LAMP-1). Cells were harvested into cold PBS at each time point and incubated on ice. At the end of the time course, cells were resuspended in PBS/0.2% BSA/0.02% NaN_3_ (FACS buffer) and labeled with antibodies against CD8α-PerCPCy5.5 and anti-CD107a-FITC (LAMP-1).

### Intracellular Cytokine Staining

Activated OT-I CTL were cultured for 5 hr at 37°C in 96-well round-bottom plates at approx. 0.5–2 × 10^6^ cells/well in complete RPMI medium containing 10% FCS, 10 U/ml recombinant human IL-2, and 5 μg/ml GolgiPlug (Becton Dickinson), with or without 1 μM of the OVA_257-264_ (SIINFEKL) or G4 (SIIGFEKL) peptides. The cells were then washed with PBS (containing 0.1% BSA and 0.02% sodium azide), labeled with anti-mouse CD8α-PerCPCy5.5 for 30 min on ice, fixed and permeabilized with the BD Cytofix/Cytoperm kit (Becton Dickinson), and labeled for intracellular cytokine production with anti-mouse IFNγ-FITC (clone XMG1.2, PharMingen). Cells were washed and analyzed on a FACSCalibur with CellQuestPro software (Becton Dickinson). In each assay, any cytokine-positive cells isolated from wells with no peptide were subtracted from the percent cytokine-positive cells incubated with peptide to yield the final value.

### Tetramer Dissociation Assay

CTL were stained with OVA_257-264_/K^b^-PE, or G4/K^b^-PE tetramers for 1 hr at room temperature and then washed with FACS buffer (PBS, 0.2% BSA, 0.02% NaN_3_). Cells were then incubated at 37°C in medium containing 50 μg/ml 28-8-6, anti-H-2D^b^/K^b^ to prevent tetramer rebinding. Cell aliquots were removed at different times into cold FACS buffer, washed, and stained with anti-CD8α-CD8α-PerCpCy5.5 for 30 min on ice. After further washing, the cells were analyzed for residual tetramer staining with a FACSCalibur (BD Biosciences).

### Immunoblotting

OT-I CTL were left untreated or stimulated with PMA (50 nM), OVA_257-264_ (1 μM), or G4 (1 μM) peptide in the presence or absence of 100 μM sodium orthovanadate for 15 min. Cell pellets were lysed for 45 min at 4°C, at 2 × 10^7^ cells/ml in lysis buffer (PBS, 2% Triton X-100, 150 mM NaCl, 50 mM Tris-Cl [pH 8.0], 1 mM MgCl_2_, complete protease inhibitor cocktail [Roche]). Cell debris and nuclei were removed by centrifugation at 13,000 rpm. 15 μl of each lysate were separated on a 10% acrylamide gel with 15 μl SDS loading buffer (Novex Tris-Glycine SDS Sample Buffer [2×], Invitrogen) after denaturation for 10 min at 95°C, before transfer onto nitrocellulose. Membranes were blocked with PBS, 0.2% Tween-20, 5% BSA, probed overnight with rabbit anti-ERK or pERK (Cell Signaling) at 4°C, washed with PBS-T, and incubated in secondary goat anti-mouse-horseradish peroxidase (HRP) for 30 min at room temperature, washed, and developed with ECL developing solution (Amersham).

### Electron Microscopy

OT-I CTL were incubated with 1 mg/ml HRP (Boehringer) to load the endocytic pathway, washed, and resuspended at ∼3 × 10^7^/ml in serum-free medium. OT-1 were mixed with equal numbers of EL4 pulsed with 1 μM OVA_257-264_ or G4 at 37°C for 4 min, diluted to ∼7 × 10^6^/ml in 24-well plates, incubated for a further 20–30 min, fixed and processed for DAB cytochemistry (Stinchcombe et al., 2001), postfixed in 1% osmium in H_2_O for 1 hr at RT, washed extensively in H_2_O and incubated overnight in 0.5% uranyl acetate in H_2_O, washed, and EPON embedded. Sections (50–150 nm) were post-stained with lead citrate and viewed with a Phillips CM100 Electron Microscope and images captured on Kodak photographic film. Negatives were scanned and recorded digitally.

### Statistical Analysis

Quantitative data are displayed as the mean of three or more independent experiments ± standard deviation. A one-way ANOVA was used to determine whether there were statistically significant differences within each experiment. If significance was found, each individual set of conditions (avidity group) was compared to the reference group (OVA_257-264_ 1 μM) via a two-tailed Student's t test. Significance was reached when p ≤ 0.05.

## Supplemental Data

Document S1. Two Figures
