# Supplementary material for: The Strength of T Cell Receptor Signal Controls the Polarization of Cytotoxic Machinery to the Immunological Synapse
Source: Immunity. 2009 Oct 16;31(4):621–31. doi: 10.1016/j.immuni.2009.08.024 (PMC2791175; doi:10.1016/j.immuni.2009.08.024)
Supplement: Document S1. Two Figures [file mmc1.pdf]

Immunity, Volume *31*

Supplemental Data

The Strength of T Cell Receptor Signal Controls the Polarization of Cytotoxic Machinery to the Immunological Synapse

Misty R. Jenkins, Andy Tsun, Jane C. Stinchcombe, and Gillian M. Griffiths

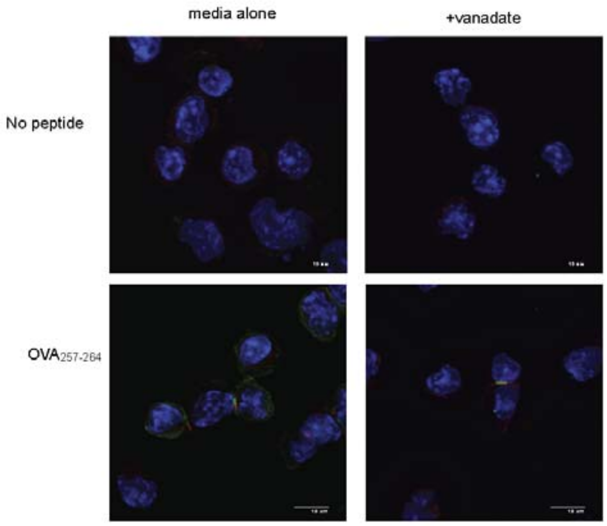

Figure S1. Orthovanadate Treatment Does Not Affect pY416 Staining in OT-1 Cells in the Absence of Peptide

Target EL4 cells were left untreated (no peptide) or pulsed with 1μM of $OVA_{257-264}$ peptide and conjugated to *in vitro* activated OT-I cells with or without 100μM sodium orthovanadate. Cells were labeled with anti-Lck (488, green), pY416 (546, red) and nuclei stained with Hoechst (blue). Confocal images of a field of conjugates shown as a projection through the xy plane. Scale bars, 10μM.

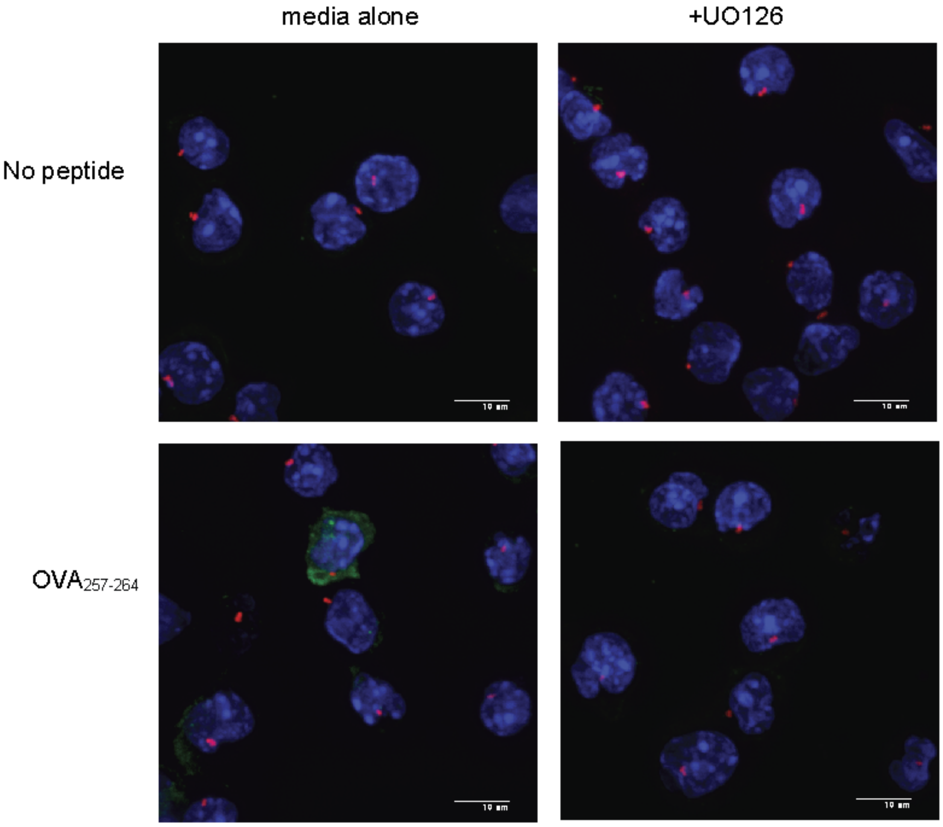

Figure S2. Preincubation of OT-1 Cells with the MEK Inhibitor, U0126, Abolishes Staining with Anti-pERK

Target EL4 cells were left untreated (no peptide) or pulsed with 1µM of $OVA_{257-264}$ peptide and conjugated to *in vitro* activated OT-I cells with or without 10µM of the MEK inhibitor, UO126. Cells were labeled with anti-pERK (488, green), γ-tubulin (546, red) and nuclei stained with Hoechst (blue). Confocal images of a field of conjugates shown as a projection through the xy plane. Scale bars, 10µM.
